# meQTL and ncRNA functional analyses of 102 GWAS-SNPs associated with depression implicate *HACE1* and *SHANK2* genes

**DOI:** 10.1186/s13148-020-00884-8

**Published:** 2020-07-02

**Authors:** Diana M. Ciuculete, Sarah Voisin, Lara Kular, Jörgen Jonsson, Mathias Rask-Andersen, Jessica Mwinyi, Helgi B. Schiöth

**Affiliations:** 1 Department of Neuroscience, Functional Pharmacology, Uppsala University, BMC, Box 593, Husargatan 3, 753124 Uppsala, Sweden; 2 Institute for Health and Sport (iHeS), Victoria University, Footscray, VIC 3011 Australia; 3 Department of Clinical Neuroscience, Center for Molecular Medicine, Karolinska Institutet, 171 76 Stockholm, Sweden; 4 Department of Immunology, Genetic and Pathology, Uppsala University, Uppsala, Sweden; 5 Institute for Translational Medicine and Biotechnology, Sechenov First Moscow State Medical University, Moscow, Russia

**Keywords:** DNA methylation, Genetics, meQTL, MicroRNA, Depression

## Abstract

**Background:**

Little is known about how genetics and epigenetics interplay in depression. Evidence suggests that genetic variants may change vulnerability to depression by modulating DNA methylation (DNAm) and non-coding RNA (ncRNA) levels. Therefore, the aim of the study was to investigate the effect of the genetic variation, previously identified in the largest genome-wide association study for depression, on proximal DNAm and ncRNA levels.

**Results:**

We performed DNAm quantitative trait locus (meQTL) analysis in two independent cohorts (total *n* = 435 healthy individuals), testing associations between 102 single-nucleotide polymorphisms (SNPs) and DNAm levels in whole blood. We identified and replicated 64 SNP-CpG pairs (p_adj._ < 0.05) with meQTL effect. Lower DNAm at cg02098413 located in the *HACE1* promoter conferred by the risk allele (C allele) at rs1933802 was associated with higher risk for depression (p_raw_ = 0.014, DNAm = 2.3%). In 1202 CD14+ cells sorted from blood, DNAm at cg02088412 positively correlated with *HACE1* mRNA expression. Investigation in postmortem brain tissue of adults diagnosed with major depressive disorder (MDD) indicated 1% higher DNAm at cg02098413 in neurons and lower *HACE1* mRNA expression in CA1 hippocampus of MDD patients compared with healthy controls (*p* = 0.008 and 0.012, respectively). Expression QTL analysis in blood of 74 adolescent revealed that hsa-miR-3664-5p was associated with rs7117514 (*SHANK2*) (p_adj._ = 0.015, mRNA difference = 5.2%). Gene ontology analysis of the miRNA target genes highlighted implication in neuronal processes.

**Conclusions:**

Collectively, our findings from a multi-tissue (blood and brain) and multi-layered (genetic, epigenetic, transcriptomic) approach suggest that genetic factors may influence depression by modulating DNAm and miRNA levels. Alterations at *HACE1* and *SHANK2* loci imply potential mechanisms, such as oxidative stress in the brain, underlying depression. Our results deepened the knowledge of molecular mechanisms in depression and suggest new epigenetic targets that should be further evaluated.

## Introduction

Depression is the leading cause of disability worldwide [1]. Twin studies have shown that depression has a heritability of 30–40% [2], and genome-wide association studies (GWASs) have uncovered many single nucleotide polymorphisms (SNPs) associated with this condition [3]. The most recent and largest genome-wide meta-analysis of depression included 807,533 individuals and identified 102 such SNPs, 87 of which were replicated in an independent cohort of 1,306,354 individuals [4]. These SNPs all showed small effect sizes, and their functional roles remain unknown. The collective profile of SNPs in each individual forms a genetic background that confers a degree of predisposition to depression. Environmental factors interact with this genetic profile to trigger depression, partly via epigenetic mechanisms, such as the regulatory action of DNA methylation (DNAm) and non-coding RNAs (ncRNA). Studying the intricate relationship between genetic variation, DNA methylation, and ncRNA expression is therefore important to understand the molecular mechanisms underpinning susceptibility to depression.

DNAm is strongly involved in neurodevelopmental processes such as synaptic formation and function [5, 6], especially during adolescence, and its perturbation can lead to psychiatric disorders [7]. CpG methylation, a process characterized by the addition of a methyl group to the fifth carbon of the cytosine ring, influences the packing of the DNA and its availability for transcription factors [8]. Typically, DNAm in gene promoters is associated with transcriptional repression while is positively associated with gene expression when located in gene bodies [9]. Site-specific DNAm is extensively influenced by the genetic background [10], age [11], sex [12], and environmental exposure, such as social stress [13, 14]. ncRNAs such as microRNAs (miRNAs) operate at the post-transcriptional level to add another layer of gene expression regulation. Increasing evidence suggests miRNAs as key modulators of synaptic plasticity and activity [15, 16] and as an important mechanism linking external stimuli to changes in brain function [17].

DNAm and miRNA expression may differ depending on the genotype at a given SNP, which then may cause alterations in cell, tissue, and whole-body physiology underlying depression. Interestingly, previous studies suggested that genetic and epigenetic variations could interact [18, 19]. This emphasizes the contribution of epigenetics to human disease, especially as alterations may be linked to non-protein-coding, disease-associated genetic variants from GWAS [20, 21].

In this study, we aimed to investigate genotype-dependent DNAm and ncRNA levels in depression, by using a multi-tissue (blood and brain) and multi-layered (genetics, epigenetics, transcriptomics) approach in different cohorts. We uncovered associations between the 102 genetic variants identified in the abovementioned GWAS and shifts in epigenetics (DNAm and ncRNA) that play a role in potential mechanisms underlying depression. First, we identified and replicated DNA methylation quantitative trait loci (meQTL) in blood and brain from two independent cohorts. We used two additional cohorts to investigate the functional consequences of those DNAm alterations at the gene expression level. Finally, we addressed association between genetic variation and ncRNA expression, using a *cis*-expression quantitative trait locus (eQTL) approach.

## Results

### Demographic characteristics of the cohorts

**Fig. 1:**
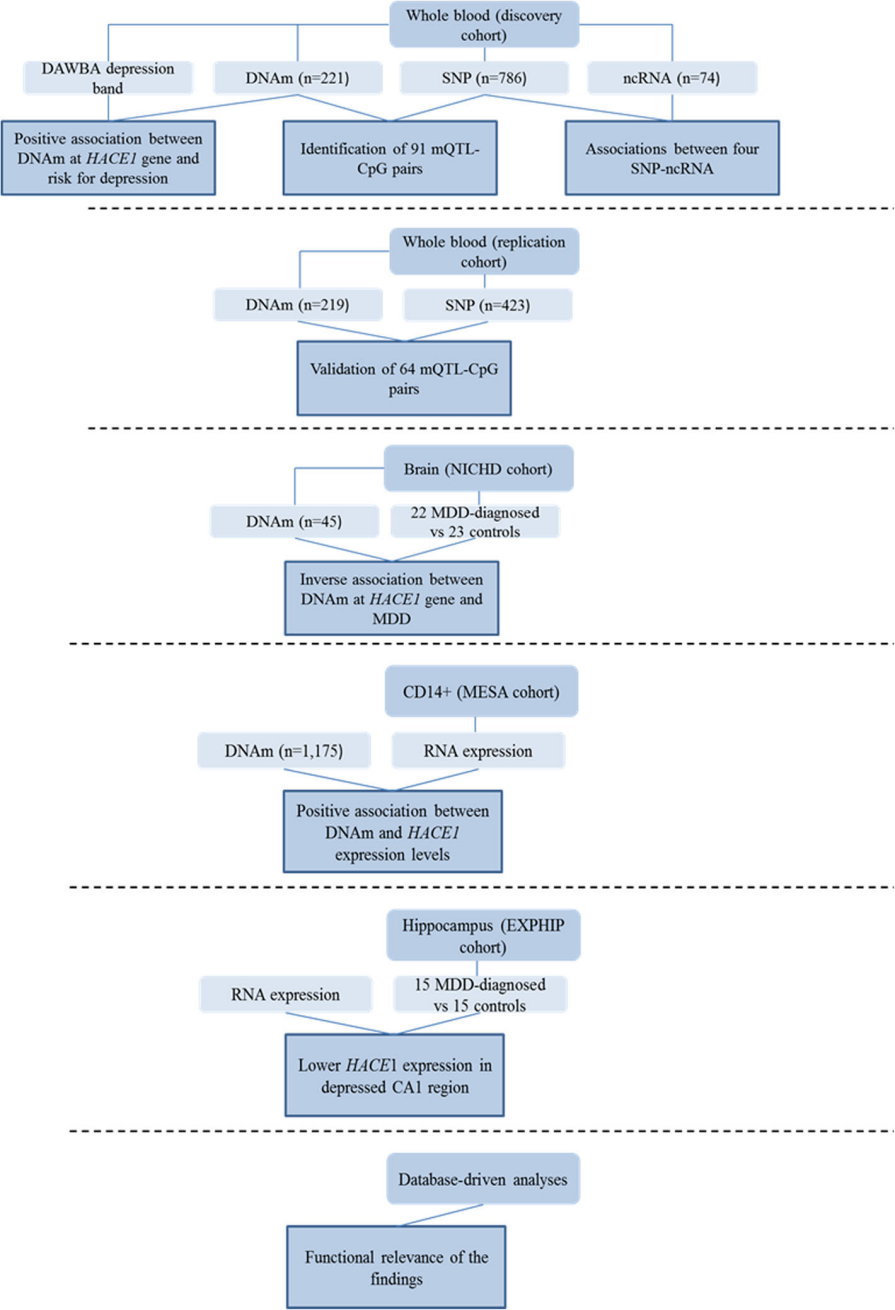
Study design and workflow diagram. Using whole blood of the discovery cohort comprising adolescents, meQTL analyses, together with associations between ncRNA levels and genotype and DNAm and depression scores were performed. The replication cohort comprised 219 adults and 64 previously identified meQTL-CpG pairs were validated in whole blood. The NICHD cohort containing 45 adult brain tissues was used to analyze the association between the MDD diagnosis and DNAm at the HACE1 gene. A positive relationship between DNAm degree and RNA expression levels of HACE1 was identified in CD14+ cells, in the MESA cohort. The EXPHIP cohort included 15 MDD-diagnosed and 15 matched controls and helped to identify lower HACE1 mRNA levels in depressed CA1 hippocampal region. The functional relevance of the findings was investigated using different bioinformatic or molecular biological software tools. DAWBA, Development and Well-Being Assessment; DNAm, DNA methylation; SNP, single-nucleotide polymorphism; ncRNA, non-coding RNA; meQTL, methylation quantitative trait locus; MDD, major depressive disorder

Our workflow is summarized in Fig. 1. Both the 216 adolescents from the discovery cohort and the 219 young adults from the replication cohort had whole blood methylome and SNP data available. Adolescents were 15.5 ± 0.63 years old, and 75% of them were female and 26% at high risk for depression, as assessed by the Development and Well-Being Assessment (DAWBA) (Table 1). The participants in the replication cohort were young adults (23.5 ± 3.3 years old), and 22% of them were females. All participants of the replication cohort, and 97.2% of the discovery cohort, were free of medication. Differences between the discovery and replication cohorts included a difference in age of approximately 8 years and in sex distribution (75% vs 22% females). Body mass index (BMI) was comparable between the two cohorts (21.9 ± 3.44 and 23.7 ± 3.03, Supplementary Figure 1). The 22 adults with major depression disorder (MDD) and the 23 matched controls from the NICHD Brain Bank of Developmental Disorders had similar age and sex distribution (Table 2).

**Table 1:** Characteristics of the adolescents from the discovery cohort

	Low-risk (*n* = 160)	High-risk (*n* = 56)	*p* value**
Male: Female	47 (19.4):113 (80.6)	6 (13.1):50 (86.9)	*0.0089*
Age (years)	15.4 ± 0.63	15.6 ± 0.65	0.15
Body mass index (kg/m^2^)	21.61 ± 3.02	22.83 ± 4.33	0.054
DAWBA level bands*			
General band	70 (19.4)	56 (100)	*6.03e-14*
Panic disorder	9 (5.6)	5 (8.9)	*0.00041*
Posttraumatic disorder	14 (2.5)	9 (16)	*5.44e-6*
Separation anxiety disorder	5 (3.1)	6 (10.7)	*2.05e-5*
Generalized anxiety disorder	30 (18.7)	23 (41.1)	*2.11e-7*
Social phobia	17 (10.6)	14 (25)	*0.016*
Obsessive-compulsive disorder	9 (5.6)	6 (10.7)	*0.028*
Conduct disorder	10 (6.2)	9 (16.1)	*7.89e-6*
Specific phobia	9 (5.6)	7 (12.5)	*0.0072*

Variables are shown as mean ± standard deviation or number (percentage)

*All listed DAWBA bands refer to individuals at high-risk (defined by DAWBA bands = 3, 4, or 5) of, e.g., general band, depression, and panic disorder

**Two-tailed Student’s *t* test for continuous variables and chi-square test for categorical variables (likelihood ratio). Significant *p* values are written in bold and italics

Individuals with depression DAWBA band risk scores <15% were considered at low-risk for depression; individuals with depression DAWBA level bands 3 (≈ 15%), 4 (≈ 50%), or 5 (> 70%) were considered at high-risk for depression

*DAWBA* Development and Well-Being Assessment

**Table 2:** Characteristics of individuals in NICHD Brain Bank of Developmental Disorders cohort

	MDD (*n* = 22)	Controls (*n* = 23)	*p* value*
Male:Female	12 (54.5):10 (45.5)	12 (52.2):11 (47.8)	1
Age (years)	33.6 ± 16.4	33.4 ± 16.2	0.95

Variables are shown as mean ± standard deviation or number (percentage).

*Student’s *t* test for continuous variables and chi-square test for categorical variables (likelihood ratio)

*MDD* major depression disorder

Expression analysis in blood cells was performed in 1202 adults in the MESA cohort, and the mean age was 60.2 ± 9.45 years old. Information about race and sex could not be extracted from the variable recoded incorporating race, sex, and study site. The 30 adults from the EXPHIP cohort had mRNA levels available in two hippocampal regions (CA1 and dentate gyrus) and were 60% males and 83% Caucasians (Table 3).

**Table 3:** Characteristics of individuals in the EXPHIP cohort

	MDD (*n* = 15)	Controls (*n* = 15)	*p* value*
Male:female	9 (60):6 (40)	9 (60):6 (40)	1
Age (years)	59.2 ± 16.7	58.4 ± 15.2	0.68
Race (C:AAm)	14 (93.3):1 (6.7)	11 (73.3):4 (26.7)	0.32

Variables are shown as mean ± standard deviation or number (percentage).

*Chi-square test for categorical variables (likelihood ratio)

*MDD* major depression disorder, *C* Caucasians, *AAm* African American

### Association of depression-related genetic risk variants with proximal DNAm in whole blood

**Fig. 2:**
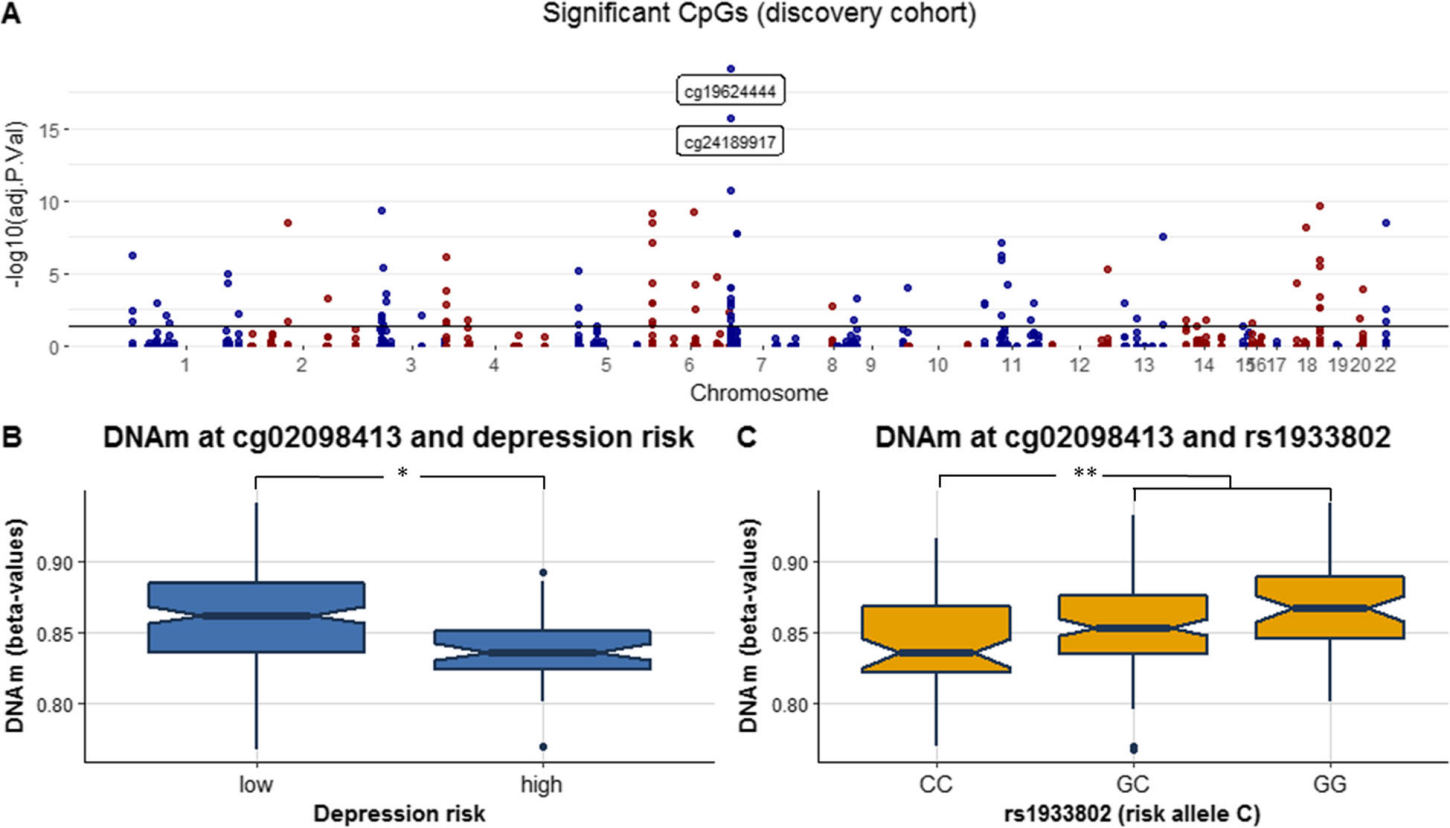
**a** Manhattan plot of the meQTL associations in whole-blood samples of 216 adolescents. The line represents the significance level at Bonferroni corrected *p* value of 0.05. **b** Blood DNAm levels (β values) at cg02098413 within the *HACE1* gene in the discovery cohort. Adolescents with depression DAWBA band risk scores below 15% were defined as “Low-risk”, while individuals with depression DAWBA level bands 3 (≈ 15%), 4 (≈ 50%), or 5 (> 70%) were assigned to the “High-risk” category. **c** Blood DNAm levels (β values) at cg02098413 within *HACE1* gene vs the presence of the risk allele (C allele) at rs1933802. *p values < 0.05, **p values < 0.005

We performed a *cis*-meQTL analysis in the discovery cohort (Table 1), using a dominant genetic model for each SNP, and controlling for age, sex, and batch. In our cohort, the minor allele frequency (MAF) of the 102 GWAS studied SNPs that were in a range similar to other Caucasian populations [4] (Supplementary Table 1). We identified 91 CpGs associated with 46 SNPs, forming 91 SNP-CpG pairs spread throughout the genome (adjusted *p* value < 0.05, Fig. 2a, Supplementary Table 1).

We then attempted to replicate our initial 91 SNP-CpG pairs in an independent cohort. Four of the 91 pairs could not be analyzed as they were excluded in the preprocessing procedure. DNA levels at the 87 remaining CpGs were linearly regressed against the previously identified meQTLs-SNPs (dominant genetic model), covarying for age and sex. In total, 64 SNP-CpG pairs were replicated in the independent cohort, with Benjamini-Hochberg (BH)-adjusted *p* values < 0.05 (Table 4, Supplementary Table 2). All but two of the 64 replicated pairs had a similar DNAm direction for SNP risk carriers in both discovery and replication cohorts. Fifty four percent of the 64 replicated CpGs were hypomethylated in carriers of the risk allele for depression (Supplementary Table 2). The rs3823624 SNP within *MAD1L1* was associated with the highest number of CpG sites (*n* = 9 CpGs). The risk allele for depression (the C allele) was associated with lower DNAm at six CpGs, with the highest effect size at the pair rs3823624-cg19624444 (6.8% DNAm difference in the discovery cohort and 8.6% in the replication cohort between T major allele carriers and C risk allele carriers).

**Table 4:** Validated meQTL-CpG pairs

meQTL SNP	Chromosome	Associated gene	No. of paired CpGs*
rs301799	1	*RERE*	2
rs1890946	1	*NRDC*	1
rs72710803	1	*AL122019*	2
rs7585722	2	*RNF103-CHMP3*	2
rs4346585	3	*SOCS5P3, ZKSCAN7-AS1*	3
rs13084037	3	*KLHDC8B*	2
rs7624336	3	*TKT*	1
rs6783233	3	*LOC101926953*	1
rs7685686	4	*HTT*	5
rs34937911	4	*SLC30A9*	1
rs60157091	5	*KIF2A*	1
rs200949	6	*HIST1H1B*	7
rs7758630	6	*LOC107984041*	1
rs1933802	6	*HACE1--[]--LIN28B*	2
rs3823624	7	*MAD1L1*	9
rs2043539	7	*TMEM106B*	2
rs7030813	9	*PAX5*	1
rs2670139	9	*DENND1A*	1
rs198457	11	*-*	4
rs7117514	11	*SHANK2*	1
rs3213572	12	*SPPL3*	1
rs1343605	13	*OLFM4--[]*	1
rs4772087	13	*STK24*	1
rs1045430	14	*AREL1*	1
rs62091461	18	*RAB27B*	1
rs7241572	18	*KCNG2*	6
rs12624433	20	*SLC12A5*	1
rs5995992	22	*RBX1---[]-EP300*	3

*Validation analysis was run by applying lm models between the methylation level and genetic dominant model, adjusting for age and sex The Benjamini-Hochberg multiple-testing adjustment was applied to the *p* values. A CpG site was paired with the meQTL SNP if the adjusted *p* value < 0.05

*meQTL* methylation quantitative trait locus, *SNP* single nucleotide polymorphism

### Lower DNAm at cg02098413 in whole blood is associated both with depression risk and with the risk allele at rs1933802 in the *HACE1* gene

We next tested whether blood DNAm at the 64 discovered and replicated CpGs was associated with depression risk in the discovery cohort. DNAm levels were available for 221 adolescents, who were categorized based on the DAWBA depression scores as “low-risk” or “high-risk” for depression. The binary variable was regressed against the 64 replicated CpGs together with age, sex, and DNAm batch as covariates (Supplementary Table 3). Higher DNAm at cg02098413 located upstream of HECT Domain and Ankyrin Repeat Containing E3 Ubiquitin Protein Ligase 1 (*HACE1*) was associated with lower depression score (p_raw_ = 0.014, odds ratio (OR) = 0.23) (Fig. 2b). Lower DNAm at cg02098413 was also associated with the risk allele at rs1933802, in both the discovery (Fig. 2c) and replication cohorts (p_adj._ = 0.0029 and 0.0063, respectively) (Supplementary Table 2). Each risk allele (C allele) was associated with a 1% decrease in DNAm at cg02098413. The MAF in adolescents at low-risk for depression was 0.54, compared with 0.42 in those at high-risk for depression.

### DNAm at cg02098413 is positively associated with *HACE1* gene expression in whole blood

DNAm levels at cg02098413 and *HACE1* mRNA levels (transcript ID: ILMN_1740217) were investigated in 1202 CD14+ samples. A 15% increase in DNAm at cg02098413 was associated with a one unit change in *HACE1* mRNA expression (*p* = 0.00060) (Supplementary Figure 2).

### Increased DNAm at cg02098413 and reduced expression of *HACE1* in the adult brain are associated with depression

**Fig. 3:**
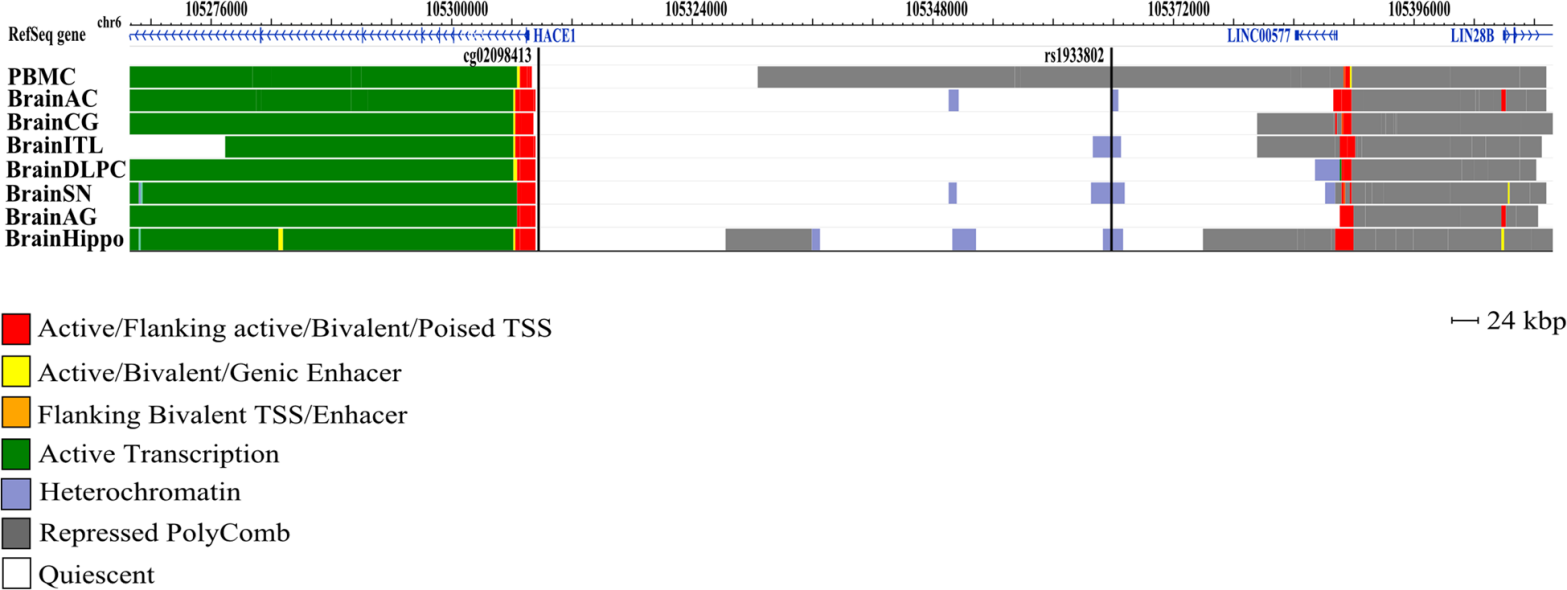
Genomic context of the CpG site associated with the depressive phenotype and genetic variant rs1933802. Genomic positions of RefSeq genes are displayed in the top part and indicated by arrows. The position of the significant CpG site is highlighted by black lines. Since analyses were performed based on data obtained in blood, chromatin marks overlapping in brain and blood cells were investigated. Chromatin states of 8 tissues downloaded from the 37/hg19 WashU Epigenome Browser are illustrated. Each functional role of a segment is indicated by a particular color. BrainAC, brain anterior caudate; BrainCG, brain cingulate gyrus; BrainHIPPO, brain hippocampus; BrainITL, brain inferior temporal lobe; BrainDPC, brain dorsolateral prefrontal cortex; BrainSN, brain substantia nigra; BrainAG, brain angular gyrus; PBMC, peripheral blood mononuclear primary cells

Exploration of chromatin states of this locus revealed that cg02098413 is located next to the active chromatin region in all seven investigated brain regions and peripheral blood mononuclear primary cells (PBMC) (Fig. 3). DNAm level at cg02098413 in blood was positively correlated with DNAm in Brodmann area (BA) 10 region (prefrontal cortex), but not to BA20 or BA7 regions. In addition, rs1933802 is a meQTL for cg02098413 in brain (*p* = 1.97 × 10^−05^, Spearman *r* = 0.19). Transcriptome data across tissues (GTEx) and at a single-cell resolution in the human analyses indicated that *HACE1* gene is highly expressed in the brain, particularly in neurons, followed by astrocytes (Supplementary Figure 3).

**Fig. 4:**
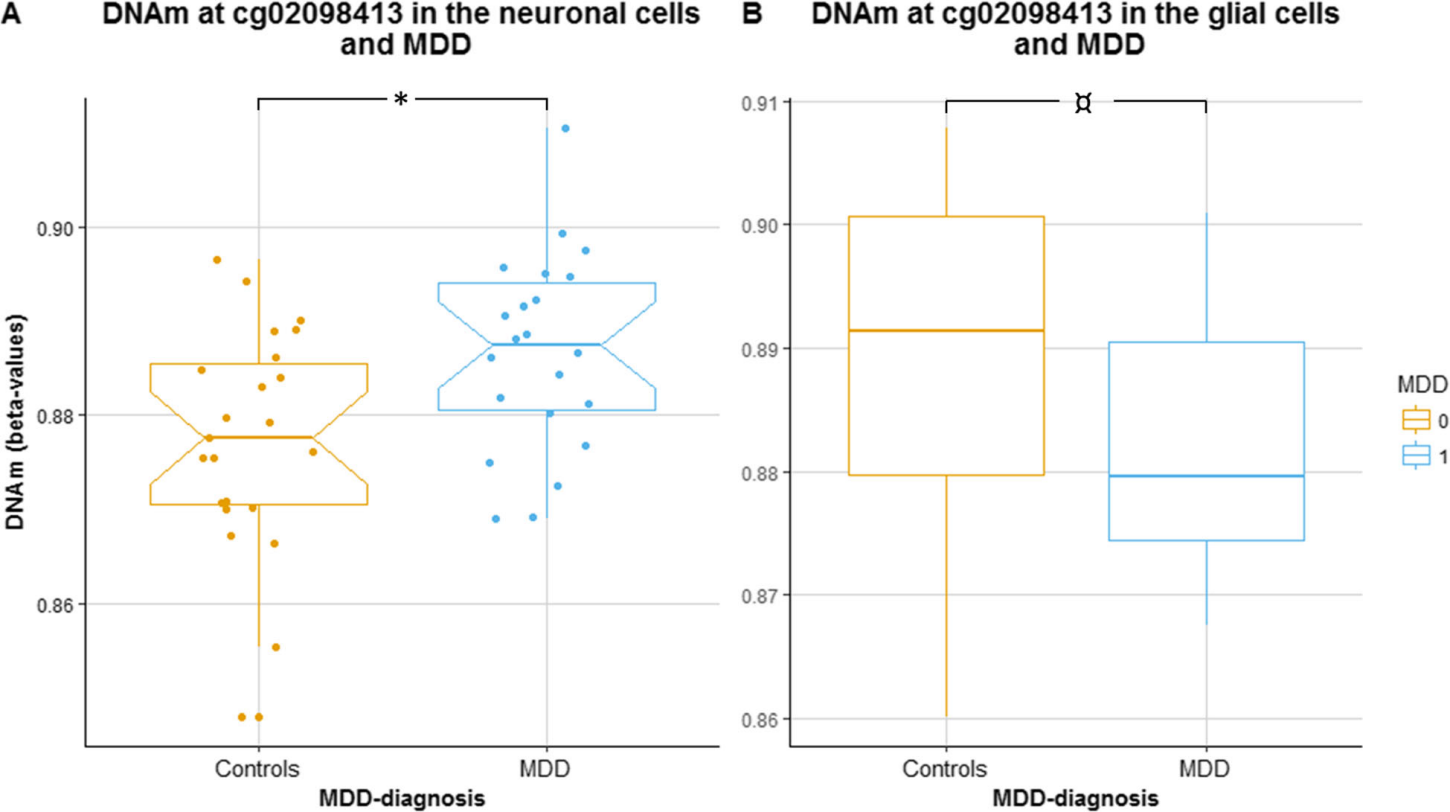
Methylation levels at cg02098413 (*HACE1*) in **a** neuronal cells and **b** glial cells of MDD diagnosed individuals and controls. *p values < 0.05, ^¤^*p* values < 0.1

We investigated the relevance of DNAm at cg02098416 in the brain of 22 adults with MDD and 23 matched controls from the NICHD Brain Bank of Developmental Disorders. DNAm was available for both neuronal and glial cells, and adjusted for age and sex. DNAm at cg02098413 in neurons was positively associated with depression (*p* = 0.008, difference of 1% between MDD and controls), and we found the opposite trend in glial cells (*p* = 0.072) (Fig. 4a, b).

We next examined *HACE1* expression in the postmortem hippocampal tissue of patients diagnosed with MDD compared with matched controls. CA1 regions from MDD individuals displayed lower mRNA levels of *HACE1* compared with controls (*p* = 0.012)*.* No difference in *HACE1* expression was identified in the dentate gyrus region (*p* = 0.6).

### Four of the 102 SNPs are eQTL for nearby ncRNA

**Fig. 5:**
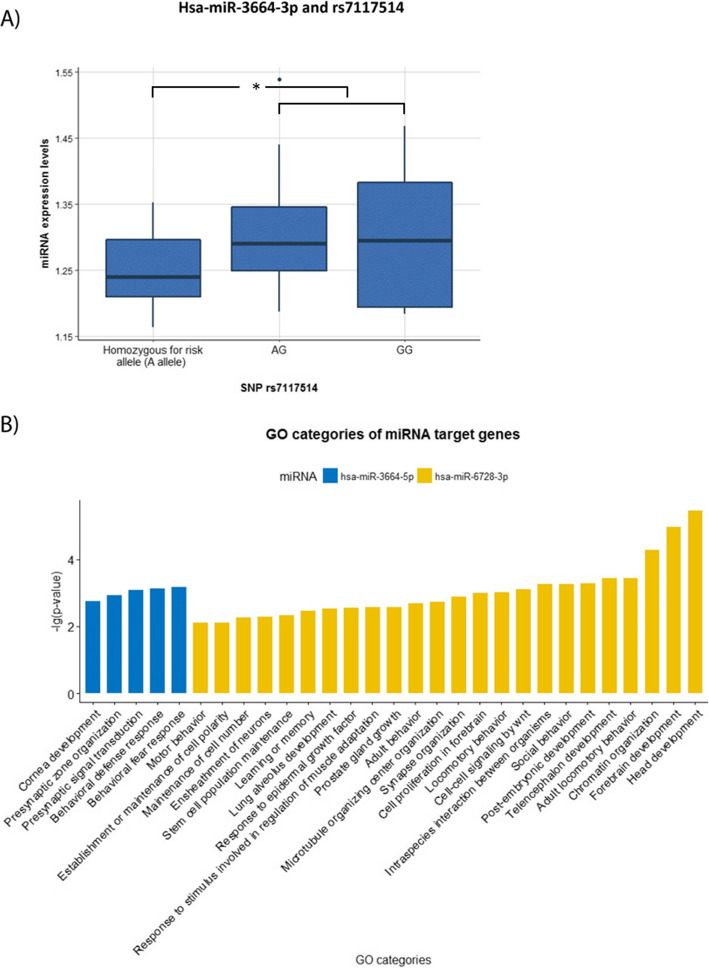
**a** Hsa-miR-3664-5p expression levels and rs7117514 genotype in blood samples of 74 adolescents. **b** Gene ontology (GO) categories for the target genes of the microRNAs hsa-miR-3664-5p and hsa-miR-6728-3p. *p values < 0.05

In addition to DNAm, we investigated the link between depression-related SNPs and expression of nearby ncRNAs (± 500 kbp window around the SNP locus) in 74 adolescents from the discovery cohort. ncRNA expression levels were regressed against the SNP, adjusting for sex and age. Out of 229 unique SNP-ncRNA tested pairs, four were significant (p_adj._ < 0.05, Table 5), the most significant association being rs7117514-miR-3664-5p (Fig. 5a).

**Table 5:** Significant SNP-ncRNA pairs after the Bonferroni multiple-testing adjustment

SNP	RefGene	Position (hg19)	ncRNA	Host gene	Bonferroni-adj. *p* value	% ncRNA mRNA levels difference
rs7117514	*SHANK2*	11:70544937	hsa-miR-3664-5p	*SHANK2*	*0.015*	5.2
rs72710803	*AL122019*	1:177428018	hsa-miR-488-5p	*ASTN1*	*0.026*	10
rs301799	*RERE*	1:8489302	hsa-miR-6728-3p	*ENO1*	*0.032*	− 8.9
rs7659414	–	4:177350956	ENSG00000201516	*WDR17*	*0.037*	− 3.9

Linear models with ncRNA expression as outcome and SNP, age, and sex as predictors. The difference in mRNA levels is shown between major allele carriers and heterozygous and homozygous for minor allele

*SNP* single nucleotide polymorphism, *ncRNA* non-coding RNA

To infer the biological relevance of miRNA expression changes, we performed gene ontology (GO) analysis of the predicted target genes for the identified miRNAs, hsa-miR-3664-5p, hsa-miR-488-5p, and hsa-miR-6728-3p. Enriched GO terms associated with hsa-miR-3664-5p target genes were behavioral fear and defense response, presynaptic signal transductions, and presynaptic active zone organization; GO term related with hsa-miR-6728-3p target genes was social behavior, synapse organization, locomotory behavior, and muscle adaptation to stimulus, learning, or memory (Fig. 5b). No significant GO terms had gene set enrichment > 10% coverage for hsa-miR-488-5p. Pathway analysis indicated neuronal system and intracellular signaling as potential processes implicating the target genes (Supplementary Table 2).

## Discussion

We addressed the potential mechanisms underlying genetic risk for depression by intersecting several molecular layers, namely, epigenetic and transcriptional, with genetic profiles. We investigated the association between genetic variations and DNAm as well as ncRNA expression in diverse datasets, including adolescents at high risk for depression and adults diagnosed with MDD. Among the replicated meQTLs, DNAm at cg02098413 located in the promoter of *HACE1* was associated with rs1933802. In addition, this CpG in whole blood and brain was associated with depression in two independent cohorts. This differential methylation could have a functional impact as *HACE1* expression levels were lower in CA1 hippocampal samples of depressed adults. Therefore, we showed cumulative evidence for rs1933802 to play a regulatory role in depression etiology via differential DNAm at cg02098413. Lastly, two GWAS SNPs were eQTL for four small ncRNAs implicated in neurobiological processes.

Approximately, half of the 102 associated SNPs with depression were found to be *cis*-meQTL, indicating that they may give susceptibility to depression via effects on local DNAm. We found that the risk allele at rs1933802 was associated with lower DNAm at cg02098413 in blood. In contrast, the risk allele was associated with higher DNAm levels in brain tissue. Additionally, DNAm at cg02098413 in whole blood was associated with depression risk, and DNAm at cg02098413 in the brain was associated with MDD. Interestingly, our data revealed the tissue specificity of genetically dependent DNAm levels in relation to MDD, i.e., lower DNAm in whole blood and higher DNAm in the prefrontal cortex. We hypothesize that rs1933802 exerts tissue- or cell type-specific effect on proximal DNAm levels, as meQTLs are coordinated by variation in transcription factor binding to specific genes, which is tissue-specific [22, 23]. Moreover, one cannot exclude the possibility of additional tissue- or cell type-specific mechanisms involved in the observed DNA methylation changes. In line with this, DNAm levels can vary within tissues, including across different brain regions [24], and we identified differential DNAm in neurons specifically, as opposed to glial cells, of MDD patients. Thus, even though we found a positive correlation between DNAm levels in BA10 within the prefrontal cortex and blood, we cannot assume the direction of the correlation between blood and the entire prefrontal cortex region.

DNAm differences at cg02098413 may have functional consequences at the cellular and molecular levels (e.g., differential transcriptional effect or splicing across tissues). The positive relationship between DNAm and *HACE1* expression we found in CD14+ cells cannot be extrapolated to a similar direction in the brain. On the contrary, the high DNAm and low *HACE1* expression observed in MDD brain, although identified in distinct brain regions and cohorts, concurs to the conventional view of anti-correlated promoter DNAm and transcriptional changes. This is supported by the diverse functions of HACE1 in cellular processes such as tumorigenesis, oxidative stress response, and neurodevelopmental disorders. Importantly, dysfunctional *HACE1* has been implicated in structural and functional brain defects [25, 26]. Previous findings in Hace1 knock-out mice showed that HACE1 is thought to modulate reactive oxygen species (ROS) via its target protein RAC1 [27]. Our results are in line with the growing body of evidence highlighting the involvement of ROS in relation to depression [28, 29] and suicidal behavior [30]. Moreover, oxidative stress, resulting from an altered production of oxidant and antioxidant reactions, can negatively affect dendritic arbors and spines. Our findings suggest that lower *HACE1* expression levels in the CA1 hippocampal region of adults diagnosed with major depression might lead to dendritic damage via increased ROS. Reduced *HACE1* levels might rely, at least partly, on DNAm as MDD-diagnosed individuals had higher DNAm levels in prefrontal cortex compared with matched controls.

In our study, the expression of three miRNAs was associated with a differential expression of their host genes. The rs7117514 SNP within *SHANK2* was associated with levels of hsa-miR-3664-5p that is located in the intronic region of the gene. Recently, hsa-miR-3664-5p was found to suppress the proliferation of gastric cancer through its target MTDH [31]. While little is known about the functional role of this miRNA, especially in a disease context, previous studies highlighted the importance of intronic miRNAs as their expression may be coordinated with the host gene’s expression [32, 33], as well as functional processes involving regulation of the hosts [34] or genes acting on related pathways [35, 36]. Hsa-miR-3664-5p host gene SHANK2 encodes the largest isoform of a family of scaffold proteins and is found abundantly at the postsynaptic site of excitatory synapses in the CNS [37]. Accordingly, SHANK2 is thought to act as an initial “organizer” of the postsynaptic density, which further recruits SHANK3. Human genetic studies have implicated aberrant expression of *SHANK2* in phenotypes such as intellectual disability, schizophrenia, and bipolar disorders [38–40]. Moreover, *SHANK2* and *SHANK3* have been shown to be causative genes for autism spectrum disorders [41, 42], possibly through the regulation of *RAC1* function [43]. Importantly, a decrease in SHANK2 protein at hippocampal CA1 synapses leads to dysregulation of *N*-methyl-d-aspartate receptors (NMDARs) in mice [44]. This is of interest since NMDAR plays a critical role in the fast synaptic glutamate neurotransmission, and its dysfunction contributes to the pathophysiology of depression [45]. A new generation of pharmacological treatments for depression includes NMDAR antagonists, such as ketamine [46].

In our study, hsa-miR-6728-3p was differentially expressed in carriers of the rs301799 allele. This intronic miRNA is located in *ENO1* that has been previously related to depression and diabetes [47]. Interestingly, GO analysis of hsa-miR-6728-3p target genes revealed processes related to motor behavior, regulation of muscle adaptation in response to stimulus, and locomotory behavior. Major depression entails significant disturbances in psychomotor function [48], such as slower motor response times [49] and decreased overall motor activity [50]. Two other GO terms relate to social behavior and learning, supporting the evidence that the genetic dependent miRNA expression could be involved in the negative response in social stimuli processing [51] and memory [52] associated with depression.

Our results are strengthened by our comprehensive bioinformatics analysis that is integrating multiple lines of evidence point at a role for *HACE1* and *SHANK2* in the pathophyology of depression. We combined five independent cohorts and multiple online tools to investigate the biological relevance of genetics, DNAm, ncRNA, and mRNA expression in depression. However, the eQTL analysis of ncRNA has not been replicated in an independent cohort. In addition, depression risk in the discovery cohort was assessed by the self-reported DAWBA questionnaire, without being validated by a clinician. The heterogeneous condition of depression is more likely to be endorsed by more individuals than those that would meet the diagnostic criteria for MDD. It should be mentioned that the DAWBA questionnaire was validated in a Brazilian sample [53]. Another limitation of our study was that age ranges and sex distribution across cohorts were different. However, while genetics appears to be especially important in regulating DNAm levels, other covarying factors were taken into account in our analyses. Importantly, the statistical models were corrected for age and sex, enabling us to adjust for the effect of age and sex on DNAm. Moreover, meQTLs were shown to be stable over the time [54], where most of age-related DNAm changes accumulate faster during the adolescence than in adulthood [55]. Sex differences in DNAm provide a potential biologic mechanism to explain the increased risk of depression in females [56]. Therefore, additional studies are required to further ascertain the findings.

## Conclusions

In conclusion, our study explores potential molecular mechanisms and biological processes underlying causal genetic variants in depression. We present a genotype-dependent DNAm at the *HACE1* gene that associates with depression in brain and whole blood across diverse datasets, specifically adolescents at risk for depression and adults diagnosed with MDD. Moreover, we found altered gene expression in neuronal cells of depressed adults, which may lead to dendrite density alteration. Furthermore, hsa-miR-3664-5p was differentially expressed in risk allele carriers of a SNP in *SHANK2*. Altered expression of miRNA and its host may contribute to the pathophysiology of depression via dysregulation of NMDARs. Together, our results underscore DNAm, ncRNA expression and genetic risk factors as an interplay modulating mechanism behind depression.

## Methods

### Discovery cohort of the meQTL analyses - DNAm, SNP imputation, ncRNA and depression risk

The discovery cohort of 221 individuals was a sample from 786 unrelated Swedish adolescents aged 17–19, recruited between 2012 and 2017 in Uppsala, Sweden. Subjects were followed up approximately 1 year after enrollment, with the aim to investigate potential psychiatric risk factors in youth. Individuals with any severe cognitive impairment were excluded. Out of the 221 adolescents, six participants were under medication for depression. Height, age, and medication intake were self-reported, but body weight and height were measured and used for BMI calculation. The risk for depression was assessed using the DAWBA web-based interview designed for individuals aged 5–17 years to generate Diagnostic and Statistical Manual of Mental Disorders IV and International Statistical Classification of Diseases and Related Health Problems-10 based diagnoses [57]. Percentage of risk for depression was categorized in six band scores, i.e., 0 (< 0.1%), 1 (≈ 0.5 %), 2 (≈ 3%), 3 (≈ 15%), 4 (≈ 50%), and 5 (> 70%). The DAWBA depression band consists of questions related to the occurrence of depression symptoms in the last 4 months (e.g., level of sadness, irritability, sleep activity, wish to die, and to what extent general mood is affected). Individuals with a score ≥ 3 are considered at high risk for depression [58].

All 786 were genotyped using the Infinium Global Screening Array at the SNP&SEQ SciLife Platform at Uppsala University, interrogating 700,078 genetic variants. SNPs were imputed as previously described [59], and all 102 selected SNPs had imputation scores > 0.8. Blood DNAm profiles of the 221 adolescents were assessed with the Illumina 450 K methylation Beadchip. Data preprocessing was performed as described in [20, 58]. It should be noted that the methylomes were profiled at two different time points within a time range of 1 year [20]. This time difference in DNAm assessment at baseline is further referred to as “batch” and taken into consideration in the meQTL analyses. We performed a *cis*-meQTL analysis of CpG sites within ± 500 kbp of each SNP.

A subset of participants (*n* = 74) had available ncRNA measurements. The RNA measurements were performed with the Affymetrix® miRNA 4.1 Array Plates, using 120 μl of the prepared biotinylated RNA from each sample. The arrays were then hybridized, washed, stained, and scanned with the GeneTitan® Multi-Channel instrument. The raw data were normalized in Expression Console provided by Affymetrix, using the robust multi-array average method [60]. A *cis*-eQTL analysis ncRNAs within ± 500 kbp of each SNP was performed.

### Replication cohort of the mQTL analyses - DNAm and SNP imputation

Participants in this study (*n* = 423) are healthy individuals of white European descent aged 18–34 years, recruited in Uppsala, Sweden. The replication cohort consisted of a subset of 219 individuals free of medication for psychiatric disorders, who had available both genetic and DNAm data in blood. Blood samples were taken in the fasted state.

All participants were genotyped using the Infinium Global Screening Array, interrogating 700,078 genetic variants. Prior to imputation, quality control steps were performed using Plink 1.9 [61]: samples were excluded based on genotyping call rate (< 95%), high heterozygosity > 2 standard deviations (SD), and missingness rate > 0.05 (*n* = 8 samples); probes were removed based on MAF (< 1%), Hardy-Weinberg equilibrium (HWE) (< 1e-6), missingness rate (> 5%), and strand ambiguousness (*n* = 219,868 probes). A total of 480,210 genotypes for 415 individuals were included in the pre-phasing approach implemented in SHAPEIT version 2.7 (r904) [62] and then imputed using the IMPUTE2 software (version 2.3.2) [63], using the 1000 Genome Project phase 3 integrated variant set as a reference (release October 2014).

Preprocessing of the DNAm data included background correction (“noob” method) [64], data normalization (all-sample mean normalization method) [65], and sample and probe exclusion. No samples were excluded based on the detection *p* < 5e-5. We excluded probes with missing *β* values, probes having ≤ 75% of samples with detection *p* < 0.01, and probes located on the sex chromosomes. Using the annotation generated by Chen et al. [66], cross-reactive probes and probes containing SNPs with minor allele frequency > 1% in European populations were removed as well. In total, 397,620 probes remained. Batch effects were corrected with the ComBat function [67]. All data processing was performed using R version 3.1.3.

### Brain DNAm profiles of MDD-diagnosed individuals and controls from the NICHD Brain Bank of Developmental Disorders

We tested associations between the newly discovered meQTLs and depression in a publicity available, processed DNAm dataset of 58 postmortem cortical tissues from 29 individuals with MDD, and 29 age- and sex-matched controls. In these samples, neuronal and glial cell proportions were measured by fluorescence activated cell sorting, and DNAm levels were separately analyzed in neurons and glia (GSE41826). To avoid confounding of DNAm signals by ethnicity, only data from Caucasian individuals were included in further analyses (*n* = 22 MDD diagnosed individuals and *n* = 23 controls) [68].

### Whole blood DNAm and mRNA expression from the Multi-Ethnic Study of Atherosclerosis (MESA)

We investigated the functional relevance of the discovered meQTLs by testing associations between DNAm levels at the meQTLs and mRNA expression in an open-access dataset (E-GEOD-56047). Methylomes were profiled with the Illumina 450 K methylation Beadchip and transcriptomes with the Illumina HumanHT-12 V4.0 expression Beadchip. Details about DNAm data preprocessing and transcriptome quality control can be found elsewhere [69]. All 1202 participants had information available on age (44–83 years old), race/ethnicity, study site, sex, DNAm/expression chip, and proportions of B-cells, T-cells, neutrophils, and natural killer cell.

### Brain mRNA levels in two hippocampal regions of MDD-diagnosed and matched controls from EXPHIP

To evaluate differential mRNA expression of target genes with depression, we compared mRNA expression levels in 15 patients with MDD with 15 age-, sex-, and race-matched controls in the dentate gyrus and CA1 subregions of the hippocampus (ArrayExpress accession E-GEOD-24095). Ten adults diagnosed with MDD were taking anti-depressants, while the controls were not under medication treatment. Data was analyzed using the 48 K human HEEBO whole-genome microarrays and further followed by normalization using linear-log normalization to stabilize the variance of low expressing genes and removal of spatial and intensity related biases, using the MAANOVA library [70].

### Functional annotation of meQTLs

We used data from the Roadmap Epigenomics Project (WashU Epigenome Browser http://epigenomegateway.wustl.edu/legacy/, human genome version hg19) to determine chromatin states at the discovered meQTL in seven brain tissues (brain angular gyrus (BrainAG), brain anterior caudate (BrainAC), brain cingulate gyrus (BrainCG), brain hippocampus (BrainHIPPO), brain inferior temporal lobe (BrainITL), brain substantia nigra (BrainSN), brain dorsolateral prefrontal cortex (BrainDLPC)) and PBMC. For easier visualization, the 18-state model was reduced to five states, i.e., active/flanking active/bivalent/poised TSS, active/bivalent/genic enhancer, flanking bivalent TSS/enhancer, active transcription, and repressed Polycomb state.

We used correlated DNAm between blood and three brain regions (BA10 (prefrontal cortex), BA20 (temporal cortex), and BA7 (parietal cortex)) in 16 individuals, using Spearman correlations in the online BECon tool https://redgar598.shinyapps.io/BECon/ [71]. More details about the preprocessing of this DNA methylation dataset are available in [71]. Moreover, the meQTL relationship was assessed in the brain using the xQTL server [72].

Single cell RNA-seq profiles from Darmanis et al. [73] were used to investigate RNA expression levels of candidate genes in cell clusters, such as neurons, astrocytes, microglia, oligodendrocytes, OPC, and endothelial cells. Expression values were log-transformed and centered using the mean.

We determined the target genes of the identified miRNAs using microT-CDS release 5.0 [74]. GO biological process categories and Reactome cell signaling pathways with a gene set enrichment > 10% were explored using Consensus path [75].

### Statistical analyses

We used linear models as implemented in the *limma* package [76] to find meQTL in the discovery cohort, with DNAm levels as the dependent variable and SNP (dominant genetic model with wild-type coded with “0” and heterozygous and homozygous for the minor allele coded with “1”), age, sex, and batch as independent variables. CpGs were deemed significant at a Bonferroni-adjusted *p* value < 0.05.

We used linear regression to find eQTL in the discovery cohort, with ncRNA expression levels as the dependent variable and SNP dominant model, age, and sex as independent variables. The Bonferroni multiple-testing adjustment was considered for each analyzed SNP.

In an independent replication cohort, linear regressions were applied on the identified CpGs against the SNP dominant model. Analyses were adjusted for age and sex. A BH-adjusted *p* value < 0.05 was considered significant. In the cohort from NICHD Brain Bank of Developmental Disorders, DNAm levels of significant CpG sites were regressed against the yes/no depression diagnosis, adjusting for age and sex. Analyses were run separately for neurons and glia cells. Using the MESA cohort, associations between DNAm and mRNA expression levels at the associated genes were investigated. DNAm data were firstly regressed against age, race, study site, gender, methylation chip, and the sample contamination with non-targeted cells, i.e., non-monocytes. The resulting residuals were included in linear regression models as an independent variable, together with expression values as dependent variables. Covariates were age, race, study site, gender, expression chip, and the sample contamination with non-targeted cells. Expression data of the EXPHIP cohort were analyzed using a two-sided binomial test, assuming a 0.5 probability threshold.

## Supplementary information

**Additional file 1: Figure S1.** BMI distribution in the discovery and replication cohorts.

**Additional file 2: Figure S2.** DNAm at cg02098413 and expression levels of *HACE1* in 1,202 CD14+ samples.

**Additional file 3: Figure S3.** RNA expression of *HACE1* in cell types of blood and brain.

**Additional file 4: Figure S1-S4.**

## Data Availability

The datasets analyzed during the current study are available from the corresponding author on reasonable request.

## Abbreviations

GWASs: Genome-wide association studies
SNP: Single nucleotide polymorphism
DNAm: DNA methylation
ncRNA: Non-coding RNA
microRNA: miRNA
meQTL: Methylation quantitative trait locus
eQTL: Expression quantitative trait locus
DAWBA: Development And Well-Being Assessment
BMI: Body mass index
MDD: Major depression disorder
MESA: Multi-Ethnic Study of Atherosclerosis
BH: Benjamini-Hochberg
HACE1: HECT Domain and Ankyrin Repeat Containing E3 Ubiquitin Protein Ligase 1
MAF: Minor allele frequency
TSS: Transcription start site
BA: Brodmann area
GO: Gene ontology
ROS: Reactive oxygen species
NMDAR: *N*-Methyl-d-aspartate receptor
HWE: Hardy-Weinberg equilibrium
