# Supplementary material for: meQTL and ncRNA functional analyses of 102 GWAS-SNPs associated with depression implicate HACE1 and SHANK2 genes
Source: Clin Epigenetics. 2020 Jul 2;12:99. doi: 10.1186/s13148-020-00884-8 (PMC7333393; doi:10.1186/s13148-020-00884-8)
Supplement: Supplementary file 4 — Additional file 4: Figure S1-S4. [file 13148_2020_884_MOESM4_ESM.docx]

Supplementary Table 1. Genotype frequencies of the 102 GWAS SNPs for depression in 216 adolescents

| **SNP** | **Position [hg19]** | **Dominant genetic model** | **No. of paired CpG*** |
| --- | --- | --- | --- |
| rs301799 | 1:8489302 | TT (72): (TC + CC) (144) | 3 |
| rs1002656 | 1:37192741 | TT (97): (TC + CC) (119) | - |
| rs1466887 | 1:37709328 | TT (79): (TC + CC) (137) | - |
| rs1890946 | 1:52342427 | TT (40): (CT + TT) (176) | 1 |
| rs10789214 | 1:67146817 | TT (80): (TC + CC) (136) | 1 |
| rs10913112 | 1:175913828 | CC (105): (CT + TT) (111) | - |
| rs72710803 | 1:177428018 | AA (181): (AC + CC) (35) | 2 |
| rs11579246 | 1:50559162 | AA (176): (AG + GG) (40) | - |
| rs2568958 | 1:72765116 | AA (96): (AG + GG) (120) | 1 |
| rs10890020 | 1:73668836 | AA (56): (AG + GG)(160) | - |
| rs113188507 | 1:80809636 | GG (115): (GA + AA) (101) | - |
| rs169235 | 1:181740924 | AA (120): (AG + GG) (96) | - |
| rs17641524 | 1:197704717 | CC (144): (CT + TT) (72) | 1 |
| rs1568452 | 2:58012833 | CC (81): (CT + TT) (135) | - |
| rs7585722 | 2:86819128 | TT (153): (TC + CC) (63) | 2 |
| rs1226412 | 2:157111313 | TT (127): (TC + CC) (89) | 1 |
| rs62188629 | 2:208044470 | GG (107): (GA + AA) (109) | - |
| rs12052908 | 2:22503044 | AA (59): (AT + TT) (157) | - |
| rs4346585 | 3:44736493 | TT (112): (TC + CC) (104) | 4 |
| rs13084037 | 3:49214066 | AA (137): (AG + GG) (79) | 2 |
| rs7624336 | 3:53244151 | GG (159): (GT + TT) (57) | 2 |
| rs141954845 | 3:61192911 | GG (85): (GA + AA) (131) | - |
| rs6783233 | 3:117509984 | CC (121): (CT + TT) (95) | 1 |
| rs1095626 | 3:157977962 | TT (81): (TC + CC) (135) | - |
| rs7659414 | 4:177350956 | AA (71): (AC + CC) (145) | - |
| rs7685686 | 4:3207142 | AA (71): (AG + GG) (145) | 5 |
| rs34937911 | 4:42110353 | TT (174): (TC + CC) (42) | 1 |
| rs45510091 | 4:123186393 | AA (201): AG (14) | - |
| rs35553410 | 4:131237381 | TT (130): (TC + CC) (86) | - |
| rs3099439 | 5:87545318 | TT (64): (TC + CC) (152) | - |
| rs10061069 | 5:93071630 | GG (141): (GC + CC) (75) | 1 |
| rs30266 | 5:103972357 | GG (94): (GA + AA) (122) | - |
| rs11135349 | 5:164523472 | CC (71): (CA + AA) (145) | - |
| rs60157091 | 5:61509655 | TT (54): (TC + CC) (162) | 3 |
| rs200949 | 6:27835435 | AA (175): (AG +GG) (41) | 8 |
| rs7758630 | 6:101387304 | TT (96): (TA + AA) (120) | 1 |
| rs9363467 | 6:66565703 | TT (90): (TC + CC) (126) | - |
| rs1933802 | 6:105365891 | GG (57): (GC + CC) (159) | 2 |
| rs2876520 | 6:142996618 | CC (61): (CG + GG) (155) | 1 |
| rs725616 | 6:147950422 | CC (85): (CT + TT) (131) | - |
| rs2029865 | 6:165121844 | TT (71): (TA + AA) (145) | 1 |
| rs3823624 | 7:2110346 | TT (136): (TC + CC) (80) | 10 |
| rs2043539 | 7:12253880 | GG (70): (GA + AA) (146) | 2 |
| rs2247523 | 7:82454404 | CC (35): (CG + GG) (181) | - |
| rs16887442 | 7:82936909 | CC (66): (CT + TT) (150) | - |
| rs58104186 | 7:109099919 | GG (62): (GA + AA) (154) | - |
| rs7807677 | 7:117502574 | TT (70): (TC + CC) (146) | - |
| rs7837935 | 8:65562019 | GG (160): (GT + TT) (56) | 1 |
| rs67436663 | 8:71347626 | GG (114): (GC + CC) (102) | - |
| rs7030813 | 9:36999369 | CC (73): (CT + TT) (143) | 1 |
| rs10817969 | 9:119731045 | TT (104): (TG + GG) (112) | - |
| rs913930 | 9:120484009 | AA (98): (AG + GG) (118) | - |
| rs2670139 | 9:126634255 | TT (118): (TC + CC) (98) | 1 |
| rs1354115 | 9:2983774 | AA (111): (AC + CC) (105) | - |
| rs1982277 | 9:11513019 | TT (141): (TC + CC) (75) | - |
| rs263645 | 9:17016503 | AA (66): (AT + TT) (150) | - |
| rs3793577 | 9:23737627 | GG (56): (GA + AA) (160) | - |
| rs59283172 | 9:25232978 | GG (176): (GA + AA) (40) | - |
| rs34653192 | 9:31124452 | GG (93): (GC + CC) (123) | 1 |
| rs997934 | 10:1795194 | CC (70): (CT + TT) (146) | - |
| rs1021363 | 10:106610839 | GG (96): (GA + AA) (120) | - |
| rs2509805 | 11:57650796 | CC (101): (CT + TT) (115) | - |
| rs198457 | 11:61471678 | CC (157): (CT + TT) (59) | 4 |
| rs58621819 | 11:65314830 | AA (155): (AT + TT) (61) | - |
| rs7117514 | 11:70544937 | AA (69): (AG + GG) (147) | 1 |
| rs61902811 | 11:113370758 | GG (94): (GA + AA) (122) | 1 |
| rs2187490 | 11:118713180 | TT (174): TG (42) | 1 |
| rs57344483 | 11:127022560 | AA (174): (AG + GG) (42) | - |
| rs1448938 | 11:30892824 | GG (69): (GA + AA) (147) | 2 |
| rs7932640 | 11:88744425 | CC (74): (CT + TT) (142) | - |
| rs78337797 | 12:23987925 | TT (179): (TG + GG) (37) | - |
| rs56314503 | 12:84465022 | TT (122): (TG + GG) (94) | - |
| rs10774600 | 12:110741356 | CC (164): (CT + TT) (52) | - |
| rs3213572 | 12:121205078 | GG (49): (GA + AA) (167) | 1 |
| rs1343605 | 13:53647048 | CC (89): (CA + AA) (127) | 1 |
| rs9592461 | 13:66941792 | AA (54): (AG + GG) (162) | - |
| rs4772087 | 13:99115041 | CC (99): (CT + TT) (117) | 2 |
| rs1409379 | 13:31907741 | TT (112): (TC + CC) (104) | 1 |
| rs9545360 | 13:80826373 | CC (134): (CA + AA) (82) | - |
| rs61990288 | 14:42074726 | GG (69): (GA + AA) (147) | 1 |
| rs1956373 | 14:60141822 | TT (113): (TG + GG) (103) | 1 |
| rs1152578 | 14:64697037 | CC (71): (CT+TT ) (145) | - |
| rs1045430 | 14:75130235 | GG (52): (GT+TT ) (164) | 1 |
| rs10149470 | 14:104017953 | AA (55): (AG + GG) (161) | - |
| rs8037355 | 15:37643831 | TT (63): (TC + CC) (153) | - |
| rs34488670 | 15:47684936 | TT (126): (TC + CC) (90) | - |
| rs7198928 | 16:7666402 | TT (90): (TC + CC) (126) | - |
| rs7200826 | 16:13066833 | CC (123): (CT + TT) (93) | - |
| rs12923444 | 16:21639710 | AA (90): (AC + CC) (126) | - |
| rs7193263 | 16:6315880 | AA (90): (AG + GG) (126) | 1 |
| rs56887639 | 16:13755530 | AA (119): (AG + GG) (97) | - |
| rs75581564 | 17:27363750 | GG (174): (GA + AA) (42) | - |
| rs12967855 | 18:35138245 | GG (94): (GA + AA) (122) | 1 |
| rs7227069 | 18:50731802 | GG (82): (GA + AA) (134) | - |
| rs12966052 | 18:52751639 | GG (148): (GC + CC) (68) | - |
| rs12967143 | 18:53099012 | CC (95): (CG + GG) (121) | - |
| rs62091461 | 18:52488672 | CC (131): (CT + TT) (85) | 1 |
| rs7241572 | 18:77580712 | GG (150): (GA + AA) (66) | 6 |
| rs33431 | 19:30939989 | TT (71): (TC + CC) (145) | - |
| rs143186028 | 20:39997404 | GG (148): (GT + TT) (68) | 1 |
| rs12624433 | 20:44680853 | GG (132): (GA + AA) (84) | 1 |
| rs5995992 | 22:41487218 | TT (108): (TC + CC) (108) | 3 |

* Validation analysis was run by applying glm models between the genetic dominant model and methylation level, adjusting for age and sex. The Benjamini-Hochberg multiple-testing adjustment was applied to the p-values. A CpG site was paired with the meQTL SNP if the adjusted p-value <0.05

Abbreviations: meQTL, methylation quantitative trait locus; SNP, single nucleotide polymorphism

Supplementary Table 2. P-values and effect sizes at the 87 SNP-CpG pairs in the discovery and replication cohorts

|  |  |  |  |  |  | Discovery cohort | | Replication cohort | |
| --- | --- | --- | --- | --- | --- | --- | --- | --- | --- |
| **SNP** | **Position[hg19]** | **Effect allele in the discovery and replication cohorts** | **Risk allele for depression in the meta-analysis** | **CpG** | **Position[hg19]** | **P_adj._*** | **logFC** | **P_adj._**** | **Effect size** |
| rs301799 | 1:8489302 | C | T | cg00546117 | 8445544 | 6.4e-07 | 0.42 | **4.0E-04** | 0.29 |
|  |  |  |  | cg01447281 | 8482688 | 0.0041 | 0.21 | **1.0E-05** | 0.32 |
|  |  |  |  | cg00120948 | 8484416 | 0.022 | 0.22 | 0.10 | 0.11 |
| rs10789214 | 1:67146817 | C | C | cg17819635 | 67217672 | 0.0084 | -0.23 | 0.53 | 0.047 |
| rs1890946 | 1:52342427 | T | T | cg20967006 | 52343399 | 0.0011 | -0.21 | **9.2E-09** | 0.32 |
| rs2568958 | 1:72765116 | G | G | cg09256413 | 72566689 | 0.028 | 0.14 | 0.38 | 0.049 |
| rs72710803 | 1:177428018 | C | A | cg19315261 | 177225875 | 1.2e-05 | 0.42 | **3.6E-08** | 0.62 |
|  |  |  |  | cg05160879 | 177322877 | 4.4e-05 | 0.41 | **5.5E-05** | 0.36 |
| rs17641524 | 1:197704717 | T | T | cg00097038 | 197382770 | 0.0073 | 0.17 | 0.87 | -0.01 |
| rs7585722 | 2:86819128 | C | T | cg08725083 | 86790843 | 3.3e-09 | 0.25 | **8.4E-08** | 0.28 |
|  |  |  |  | cg09273112 | 86732412 | 0.024 | 0.21 | **7.9E-04** | 0.24 |
| rs1226412 | 2:157111313 | C | C | cg05987564 | 156981619 | 0.00059 | 0.19 | 0.30 | 0.052 |
| rs4346585 | 3:44736493 | C | T | cg10265740 | 44622864 | 5.1e-10 | -0.49 | **5.5E-11** | -0.48 |
|  |  |  |  | cg09333631 | 44802603 | 0.0081 | 0.30 | **7.4E-08** | 0.46 |
|  |  |  |  | cg21672276 | 44754071 | 0.010 | 0.20 | **6.5E-05** | 0.30 |
|  |  |  |  | cg02306612 | 44666376 | 0.025 | 0.12 | 0.87 | 0.0042 |
| rs13084037 | 3:49214066 | G | A | cg16083429 | 49237499 | 4.1e-06 | 0.27 | **2.6E-08** | 0.29 |
|  |  |  |  | cg01454592 | 49236799 | 0.036 | 0.23 | **1.7E-07** | 0.31 |
| rs7624336 | 3:53244151 | T | G | cg04981430 | 53213814 | 0.00029 | 0.17 | **0.026** | 0.12 |
|  |  |  |  | cg16894138 | 53270349 | 0.00098 | -0.22 | 0.28 | -0.068 |
| rs6783233 | 3:117509984 | T | C | cg07910724 | 117604410 | 0.0076 | 0.21 | **3.0E-04** | 0.28 |
| rs7685686 | 4:3207142 | G | G | cg02754929 | 3295735 | 7.9e-07 | -0.58 | **1.1E-11** | -0.70 |
|  |  |  |  | cg00810483 | 3375143 | 1.7e-04 | -0.22 | **4.9E-07** | -0.27 |
|  |  |  |  | cg22655196 | 3374908 | 1.3e-03 | -0.21 | **3.7E-07** | -0.26 |
|  |  |  |  | cg13731523 | 3047189 | 2.2e-02 | 0.23 | **2.0E-03** | 0.16 |
|  |  |  |  | cg14003022 | 3043018 | 2.7e-02 | 0.31 | **3.9E-03** | 0.23 |
| rs34937911 | 4:42110353 | C | C | cg08702941 | 42155307 | 0.015 | 0.26 | **5.0E-03** | 0.25 |
| rs60157091 | 5:61509655 | C | C | cg11891522 | 61605246 | 6.2E-06 | 0.26 | **8.2E-05** | 0.23 |
|  |  |  |  | cg01131866 | 61601528 | 0.0023 | 0.16 | 0.33 | 0.058 |
|  |  |  |  | cg08882271 | 61708403 | 0.039 | 0.14 | 0.31 | 0.057 |
| rs10061069 | 5:93071630 | C | C | cg25342875 | 93076909 | 0.048 | -0.14 | 0.81 | 0.012 |
| rs200949 | 6:27835435 | G | G | cg03597347 | 28058908 | 7.8E-10 | 0.78 | **1.2E-08** | 0.75 |
|  |  |  |  | cg11517269 | 28058788 | 3.3E-09 | 0.60 | **5.3E-07** | 0.59 |
|  |  |  |  | cg12623302 | 28058801 | 7.6E-08 | 0.64 | **7.9E-07** | 0.63 |
|  |  |  |  | cg09156207 | 28058723 | 5.1E-05 | 0.63 | **2.8E-04** | 0.50 |
|  |  |  |  | cg20715953 | 27802816 | 0.0011 | 0.25 | **0.041** | 0.14 |
|  |  |  |  | cg18105139 | 28058855 | 0.0011 | 0.46 | **1.1E-07** | 0.59 |
|  |  |  |  | cg12351042 | 27879748 | 0.023 | 0.23 | 0.052 | 0.13 |
|  |  |  |  | cg23281280 | 28129358 | 0.039 | 0.42 | **3.7E-07** | 0.61 |
| rs7758630 | 6:101387304 | A | A | cg09795085 | 101329168 | 5.6E-10 | 0.28 | **9.2E-10** | 0.27 |
| rs1933802 | 6:105365891 | C | C | cg14246568 | 105388152 | 6.8E-05 | -0.39 | **7.9E-04** | -0.26 |
|  |  |  |  | cg02098413 | 105308734 | 0.0029 | -0.23 | **5.4E-03** | -0.19 |
| rs2876520 | 6:142996618 | G | C | cg18040813 | 143381504 | 1.9E-05 | -0.20 | Not available |  |
| rs2029865 | 6:165121844 | A | A | cg08510178 | 165062069 | 0.00046 | 0.20 | 0.70 | -0.031 |
| rs3823624 | 7:2110346 | C | C | cg19624444 | 2048496 | 6.6E-20 | -0.96 | **1.1E-34** | -1.22 |
|  |  |  |  | cg24189917 | 1970922 | 1.9E-16 | -0.39 | Not available |  |
|  |  |  |  | cg02825527 | 2087842 | 1.9E-11 | -0.26 | **4.8E-05** | -0.22 |
|  |  |  |  | cg02272667 | 1922865 | 9.1E-05 | 0.13 | **4.1E-03** | 0.082 |
|  |  |  |  | cg06100570 | 2078987 | 9.9E-05 | -0.22 | **2.9E-05** | -0.18 |
|  |  |  |  | cg02743256 | 2109352 | 0.00055 | -0.22 | **6.5E-05** | -0.22 |
|  |  |  |  | cg17179570 | 1936389 | 0.0011 | -0.24 | **0.043** | -0.091 |
|  |  |  |  | cg11196182 | 1989411 | 0.0021 | 0.36 | **0.044** | 0.16 |
|  |  |  |  | cg12366968 | 1883875 | 0.0086 | 0.17 | **1.6E-03** | 0.15 |
|  |  |  |  | cg12376829 | 1915362 | 0.017 | -0.19 | **0.0021** | -0.16 |
| rs2043539 | 7:12253880 | A | G | cg21238440 | 12159717 | 1.7E-08 | 0.24 | **0.030** | 0.11 |
|  |  |  |  | cg19800032 | 12134188 | 2.0E-08 | 0.38 | **9.3E-07** | 0.37 |
| rs7837935 | 8:65562019 | T | T | cg05740045 | 65499925 | 0.0018 | 0.23 | 0.29 | 0.40 |
| rs34653192 | 9:31124452 | C | C | cg14184164 | 30688980 | 0.019 | 0.10 | 0.52 | 0.044 |
| rs7030813 | 9:36999369 | T | C | cg13738729 | 36989126 | 0.00059 | 0.18 | **0.0028** | 0.15 |
| rs2670139 | 9:126634255 | C | T | cg00407468 | 126692954 | 0.00010 | 0.14 | **0.0010** | 0.13 |
| rs1448938 | 11:30892824 | A | G | cg14844989 | 31128819 | 0.0011 | -0.29 | 0.31 | -0.10 |
|  |  |  |  | cg06552810 | 31128659 | 0.0014 | -0.32 | 0.16 | -0.15 |
| rs198457 | 11:61471678 | T | T | cg25599065 | 61443130 | 9.0E-08 | 0.42 | **6.4E-04** | 0.28 |
|  |  |  |  | cg04339947 | 61443051 | 5.9E-07 | 0.39 | **0.0011** | 0.24 |
|  |  |  |  | cg09147119 | 61425221 | 1.2E-06 | -0.58 | **2.3E-06** | -0.47 |
|  |  |  |  | cg04709703 | 61449897 | 0.0074 | 0.30 | **0.0020** | 0.21 |
| rs7117514 | 11:70544937 | G | A | cg08288223 | 70563130 | 5.8E-05 | -0.24 | **5.9E-05** | -0.24 |
| rs61902811 | 11:113370758 | A | A | cg05590274 | 113262624 | 0.018 | -0.17 | 0.077 | -0.10 |
| rs2187490 | 11:118713180 | G | T | cg15880738 | 118215111 | 0.0010 | 0.20 | Not available |  |
| rs3213572 | 12:121205078 | A | G | cg02419362 | 121203947 | 5.3E-06 | 0.32 | **1.2E-06** | 0.25 |
| rs1409379 | 13:31907741 | C | C | cg17180284 | 31783982 | 0.0013 | -0.14 | Not available |  |
| rs1343605 | 13:53647048 | A | C | cg16779498 | 53442029 | 0.015 | 0.29 | **7.9E-04** | 0.28 |
| rs4772087 | 13:99115041 | T | C | cg09504131 | 99174874 | 3.1E-08 | 0.14 | **0.0012** | 0.10 |
|  |  |  |  | cg03701759 | 99174929 | 0.03 | 0.13 | 0.30 | 0.047 |
| rs61990288 | 14:42074726 | C | A | cg13526007 | 42076709 | 0.019 | 0.18 | 0.21 | 0.10 |
| rs1956373 | 14:60141822 | G | T | cg00708844 | 60558038 | 0.043 | 0.11 | 0.70 | 0.017 |
| rs1045430 | 14:75130235 | T | T | cg06998765 | 75389619 | 0.017 | 0.19 | **8.3E-05** | -0.29 |
| rs7193263 | 16:6315880 | G | A | cg02008627 | 6318632 | 0.031 | -0.09 | 0.40 | -0.036 |
| rs12967855 | 18:35138245 | A | G | cg07300558 | 35145354 | 4.5E-05 | 0.19 | 0.080 | 0.089 |
| rs62091461 | 18:52488672 | T | T | cg12377874 | 52495403 | 7.1E-09 | 0.53 | **2.9E-05** | 0.31 |
| rs7241572 | 18:77580712 | A | G | cg20788561 | 77586854 | 2.1E-10 | -0.68 | **4.3E-10** | -0.64 |
|  |  |  |  | cg26740109 | 77585427 | 1.2E-06 | -0.53 | **9.3E-10** | -0.48 |
|  |  |  |  | cg20450689 | 77586146 | 2.9E-06 | -0.57 | **9.7E-10** | -0.65 |
|  |  |  |  | cg06705017 | 77552401 | 0.00040 | 0.28 | **0.024** | 0.16 |
|  |  |  |  | cg10606486 | 77608995 | 0.0024 | 0.23 | **0.026** | 0.14 |
|  |  |  |  | cg24856264 | 77612152 | 0.0026 | 0.35 | **0.022** | 0.20 |
| rs143186028 | 20:39997404 | T | G | cg13949816 | 40251775 | 0.014 | 0.12 | 0.52 | -0.033 |
| rs12624433 | 20:44680853 | A | G | cg20003638 | 44687729 | 0.00012 | 0.21 | **1.3E-06** | 0.23 |
| rs5995992 | 22:41487218 | C | T | cg07519229 | 41486834 | 3.2E-09 | -0.37 | **9.6E-10** | -0.40 |
|  |  |  |  | cg18501942 | 41414082 | 0.0032 | -0.15 | **1.8E-05** | -0.20 |
|  |  |  |  | cg01849789 | 41697278 | 0.021 | -0.14 | **1.1E-05** | -0.19 |

In both the discovery and replication cohorts, the effect allele was the minor allele. The effect direction (logFC and effect size) was observed for the effect allele.

*Bonferroni-adjusted p-values ** Benjamini-Hochberg –adjusted p-values < 0.05 are in bold

Abbreviations: SNP, single nucleotide polymorphism; logFC, log fold change

Supplementary Table 3. CpGs associated with depression risk in the discovery cohort

| **CpG** | **Associated gene** | **P_raw_** | **OR** |
| --- | --- | --- | --- |
| **cg02098413** | *HACE1* | 0.014 | 0.23 |
| **cg12377874** | *RAB27B* | 0.014 | 0.48 |

The associated gene of the CpG was identified using Price et al. annotation (1). OR, odds ratio

Supplementary Table 4. Reactome pathways of target genes by hsa-miR-3664-5p and hsa-miR-6728-3p

| **Pathway name** | **p-value** | **q-value** |
| --- | --- | --- |
| **hsa-miR-3664-5p** |  |  |
| PTEN Regulation | 2.13E-05 | 0.0031 |
| Post-transcriptional silencing by small RNAs | 2.30E-05 | 0.0031 |
| Competing endogenous RNAs (ceRNAs) regulate PTEN translation | 4.50E-05 | 0.0037 |
| Regulation of PTEN mRNA translation | 4.50E-05 | 0.0037 |
| **hsa-miR-6728-3p** |  |  |
| Neuronal System | 8.05E-05 | 0.043 |
| Intracellular signaling by second messengers | 0.00015 | 0.043 |
| PIP3 activates AKT signaling | 0.00016 | 0.043 |
| Negative regulation of FGFR2 signaling | 0.00026 | 0.046 |

1. Price ME, Cotton AM, Lam LL, Farre P, Emberly E, Brown CJ, et al. Additional annotation enhances potential for biologically-relevant analysis of the Illumina Infinium HumanMethylation450 BeadChip array. Epigenetics & chromatin. 2013;6(1):4.
